# Peptide-Decorated Supramolecules for Subcellular Targeted Cancer Therapy: Recent Advances

**DOI:** 10.3389/fchem.2020.00824

**Published:** 2020-10-28

**Authors:** Hua Jin, Xiao Lin, Mengyue Gao, Liao Cui, Yun Liu

**Affiliations:** Guangdong Key Laboratory for Research and Development of Natural Drugs, Guangdong Medical University, Zhanjiang, China

**Keywords:** supramolecules, functional peptides, subcellular targeted, cancer therapy, supramolecular cancer nanomedicine

## Abstract

Binding small molecules through non-covalent molecular forces affords supramolecules, such as hydrogen bonds, with electrostatic, π-π interactions, van der Waals forces, and hydrophobic effects. Due to their good biocompatibility, low immunogenicity, and biodegradability, supramolecules have been intensely studied as multifunctional drug delivery platforms in targeted cancer therapy. In consideration of the defective therapeutic efficacy induced by simply transporting the therapeutic agents into tumor tissues or cancer cells instead of subcellular organelles, research is progressing toward the development of subcellular targeted cancer therapy (STCT) strategies. STCT is one of the most recent developments in the field of cancer nanomedicine. It is defined as the specific transportation of therapeutic agents to the target organelles for cancer treatment, which makes therapeutic agents accumulate in the target organelles at higher concentrations than other subcellular compartments. Compared with tumor-targeted and cancer-cell-targeted therapies, STCT exhibits dramatically improved specificity and precision, diminished adverse effects, and enhanced capacity to reverse multidrug resistance (MDR). Over the past few decades, peptides have played increasingly essential roles in multi-types of tumor-targeted drug delivery systems. Moreover, peptide-mediated STCT is becoming an emerging approach for precision cancer therapy and has been used in various cancer treatments, such as photothermal therapy (PTT), photodynamic therapy (PDT), chemotherapy, gene therapy, and non-drug-loaded nanoassemblies. In this review, we will focus on recent innovations in the variety of peptides used in designing peptide-decorated supramolecules for cell-membrane-, mitochondria-, and nucleus-localized STCT.

## Introduction

In the past few decades, the rapid development of cancer nanomedicine has been focusing on overcoming challenges encountered by conventional medicines, such as low therapeutic efficacy, poor targetability, adverse side effects, and MDR (Sun et al., 2014). The employment of supramolecules in cancer treatment gives the definition of supramolecular cancer nanomedicine (Cui and Xu, 2017; Feng et al., 2017). Supramolecules are generated by the well-ordered self-assembly of small molecules through non-covalent molecular forces, such as hydrogen bonds, enabling them to have electrostatic, π-π interactions, van der Waals forces, and hydrophobic effects (Webber et al., 2016). In terms of their good biocompatibility, low immunogenicity, and biodegradability, supramolecules have been widely developed as multifunctional drug delivery platforms in the field of supramolecular cancer nanomedicine (Yao et al., 2018; Chen R. et al., 2019). Moreover, tremendous progress has been made in developing various therapeutic strategies over the past two decades, including chemotherapy, gene therapy, PDT, PTT, and non-drug-loaded nanoassemblies (Dong et al., 2018; Kim et al., 2018; Cheng H. et al., 2019c; He H. et al., 2019; Zou et al., 2020). Hence, significant advancements in supramolecular cancer nanomedicine and therapeutic strategies have facilitated the development of novel therapeutic nanoplatforms to resolve challenging issues of conventional medicines.

In cancer cells, all types of subcellular organelles are indispensable, which play fundamental roles in critical cellular functions. Recently, supramolecules have been applied for STCT (Gao et al., 2019; Nurunnabi et al., 2019; Guo et al., 2020). Studies indicate that the specific transportation of therapeutic agents to the target subcellular compartments, for instance, cell membrane, mitochondria, and nucleus, can be achieved through using supramolecular nanoplatforms (Song et al., 2015; Zhong et al., 2015; Deng et al., 2020). In contrast to conventional cancer nanomedicines, supramolecule-mediated STCTs exhibit some unique merits (Chen et al., 2018). Firstly, the organelle-specific delivery of therapeutics to the sites of action in cancer cells is capable of affording an optimal dose administration. Consequently, the adverse side effects caused by off-target drug delivery and high dose can be dramatically relieved. In addition, it could provide a greatly promising approach to circumvent MDR via the inhibition of drug efflux through the physical barriers of certain organelles. Namely, it exhibits dramatically improved specificity, enhanced therapeutic efficacy, and better precision over conventional cancer nanomedicines. Due to their small sizes, good biocompatibility, low cost, and various functions, functional peptides are particularly appropriate for supramolecules-mediated STCT. In this mini review, we summarize the latest development of functional peptide-decorated supramolecules for STCT in the last 5 years, with an emphasis on their outstanding performance for modulating various therapeutic strategies.

## Functional Peptide-Based Supramolecules for STCT

According to their functions in supramolecules-mediated STCT, functional peptides can be classified into three categories: tumor targeting peptides, tumor-environment-responsive peptides, and other functional peptides (Rong et al., 2020). In accordance with the specific localization sites, tumor targeting peptides can be grouped into three main types, including tumor-environment targeting peptides, cancer cells targeting peptides, and subcellular targeting peptides. Through the employment of tumor-environment-responsive peptides, the supramolecular nanoplatforms could respond to the characteristic stimuli of the tumor microenvironment for improved cellular internalization or controlled drug release, for instance, mild acidity, elevated temperature, high enzyme concentration, hypoxia, and imbalanced redox status. To enhance the therapeutic efficacy, other functional peptides can be employed to construct the supramolecular nanoplatforms, such as cell penetrating peptides (CPP) and therapeutic peptides. In summary, the utilization of functional peptides is able to greatly enhance their therapeutic efficacy to the subcellular locations in the course of cell membrane-, mitochondria-, and nucleus-targeted cancer therapy.

### Cell Membrane-Targeted Cancer Therapy

**Figure 1:**
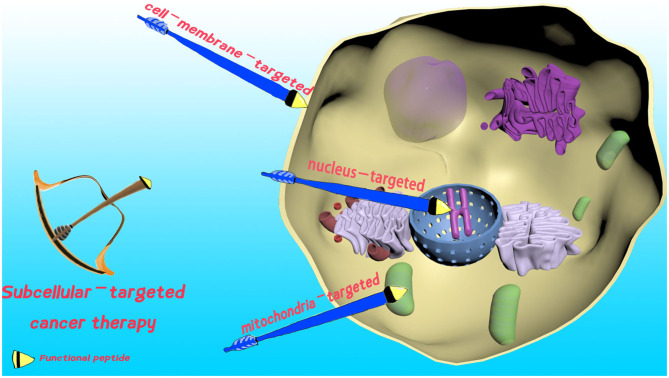
Schematic presentation of functional peptide-decorated supramolecules for cell membrane-, mitochondria-, and nucleus-targeted cancer therapy.

The cell membrane defines the borders of cells, and plays an essential role in maintaining cell integrity, cell internalization, and protecting living cells (Tani et al., 1978). Undoubtedly, damage of the cell membrane could dramatically increase the permeability. Consequently, it can lead to enhanced cellular uptake of therapeutic agents for improved therapeutic efficacy, or even cell death (see Figure 1). Hence, cell membrane-localized PTT (Chen P. et al., 2020), PDT (Ma et al., 2019), chemotherapy (Zhang C. et al., 2018; Wang et al., 2019), and non-drug-loaded supramolecular nanoassemblies (Hu et al., 2017) can be used to address MDR, avoid cell barriers, and enhance therapeutic efficacy in STCT.

As a type of noninvasive therapeutic approach, cell death in cancer cells could be efficiently triggered by PTT at a recommended target temperature range of 41–48°C (Fernandes et al., 2020), which can be generated by the activation of photothermal agents via near infrared (NIR) laser irradiation. Compared with other targeted PTTs, cell membrane-targeted PTT can avoid the inducible heat resistance of cancer cells caused by heat shock protein 70. In a recent study, Chen et al. designed the chimeric peptide-based dual-usage NIR fluorescence probe C_16_-CARK, which was used both for cell membrane-targeted fluorescence imaging and PTT (Chen P. et al., 2020). The Arg-Arg-Lys (RRK) segment endows the system with a cell membrane-targeting property, while the alkyl chain C_16_ facilitates C_16_-CARK to insert into the cell membrane with a long retention time (>4 h). Subsequently, the Cy5.5 moiety produced heat upon NIR laser irradiation to destroy the cell membrane *in situ*, and lead to cell death without the occurrence of heat resistance.

Nowadays, PDT has attracted a massive amount of research attention due to its minimal invasiveness, tunability, targetability, and few side effects (Dai et al., 2019; Chen J. et al., 2020). Upon the laser irradiation of photosensitizers (PSs) in the presence of the molecule oxygen, the produced reactive oxygen species (ROS) result in the apoptosis of cancer cells. In reality, PDT drugs were approved for bladder cancer, skin cancer, esophagus cancer, and lung cancer (Chilakamarthi and Giribabu, 2017; Kessel, 2019). However, it is still a challenging task to fulfill long-term anchoring of PSs onto cell membranes for cell membrane-targeted PDT, due to cellular uptake and endocytosis. Zhang's group pioneered in this field by using chimeric peptides as a cell membrane anchoring strategy (Liu et al., 2017). Their early research employed the chimeric peptide-modified C_16_-PRP-DMA, which can undergo charge reversal in a mildly acidic tumor environment. After the insertion onto the cell membrane through the C_16_ chain and chimeric peptide, *in situ* PDT took place upon the lase irradiation of PS protoporphyrinIX (PpIX). In their following research, the chimeric peptide strategy was further expanded to engineer cracked cancer cell membranes (CCCM) and exosomes, respectively (Qiu et al., 2018; Cheng H. et al., 2019b). Accordingly, the cell membranes were seriously damaged by the *in situ* occurred ROS, which induced apoptosis of cancer cells.

Despite of new therapeutic options, chemotherapy remains a cornerstone in cancer treatment. To overcome drug efflux induced MDR through the perturbation of the tumor membrane, cell membrane-targeted chemotherapy has recently drawn intensive research attention (Zhang X. et al., 2018). Zhang's group further used the chimeric peptide concept mentioned above for chemotherapy (Zhang C. et al., 2018). The therapeutic system loaded with doxorubicin (DOX) was denoted as CTGP. Upon arriving at tumor sites, the chimeric peptides anchored onto the cell membrane and generated self-assembled networks *in situ*, which strictly limited DOX efflux to obtain 49-fold greater anti-MDR ability than free DOX. Similarly but different, Xu's group perturbed the cell membrane to cause permeability enhancement of DOX through the recognition-reaction-aggregation (RRA) strategy (Wang et al., 2019).

Non-drug-loaded peptide nanoassemblies for cancer targeted therapy have been clearly proven by recent research (Hu et al., 2014). Particularly, it is not the therapeutic agents, the peptide supramolecular nanoassemblies, themselves that eliminate the cancer cells through physical disturbance (Lu et al., 2016). Non-drug-loaded peptide nanoassemblies for cell membrane-targeted cancer therapy can be conducted by the formation of an artificial extracellular matrix (AECM), which severely restricted cancer invasion and metastasis. For example, Wang's group developed a laminin-mimic peptide BP-KLVFFK-GGDGR-YIGSR, which specifically targeted cell membranes, transformed them into a supramolecular network, and formed AECM for the inhibition of cancer migration and metastasis (Hu et al., 2017).

### Mitochondria-Targeted Cancer Therapy

Mitochondria have become the hottest subcellular target for precise cancer therapy (Wu et al., 2018; Lee and Cho, 2019). Mitochondrion take charge of ATP production and mitochondrial apoptosis. In consideration of its remarkably significant biological importance, mitochondria-targeted transportation of therapeutics to efficiently modulate its biological function can provide new strategies for STCT (see Figure 1). However, the efficiently specific transportation of therapeutic agents to mitochondria is still a challenging task, due to the extremely high negative transmembrane potential (~-180 mV, Milane et al., 2015). Thus, cancer therapy is often mediated by the use of mitochondria-targeting moieties, including mitochondria-targeting signal peptides (MTSs), Szeto–Schiller (SS) peptides, mitochondria-penetrating peptides, (MPPs), and cationic triphenylphosphonium (TPP, Jean et al., 2016).

In consideration of the fact that mitochondria are the major organelles for ROS generation, the overexpression of mitochondrial ROS by mitochondria-targeted PDT could result in severe mitochondrial dysfunction. Therefore, mitochondria-targeted transportation of PSs via supramolecules has drawn considerable research efforts (Peng et al., 2020). Among the mitochondria-targeting molecules mentioned above, the pro-apoptosis peptide (KLAKLAK)_2_ is the most utilized ligand for mitochondria-targeted PSs delivery (Han et al., 2016b; Cheng H. et al., 2019a). Plenty of evidence indicates that (KLAKLAK)_2_ not only localizes therapeutics to mitochondria but also functions as a bio-drug itself, which could destroy the mitochondrial membrane and initiate apoptosis (Agemy et al., 2011). Zhang's group designed an effective drug self-delivery system PpIX-PEG-(KLAKLAK)_2_ to conduct mitochondria-targeted PDT (Han K. et al., 2015). Here, (KLAKLAK)_2_ acts both as the targeting segment and therapeutic agent to synergistically enhance PDT. Consequently, chemo/photodynamic synergistic therapy is realized in this system.

According to their mechanisms of action, almost all of the chemical drugs need to localize to specific organelles to elicit their pharmacological activities, including DOX, paclitaxel (PTX), camptothecin (CPT), cisplatin, and peptides. For this reason, mitochondria localization would achieve an optimal therapeutic efficiency and diminished side effects (Laws et al., 2018; Zhang et al., 2019; Zhu et al., 2019). As an ideal mitochondria-targeting cytotoxic drug, (KLAKLAK)_2_ has been widely used for mitochondria-targeted chemotherapy (Chen S. et al., 2019; Cong et al., 2019). In 2019, Wang's group developed an ROS-responsive polymer-peptide conjugate (PPC) with (KLAKLAK)_2_ for tumor therapy (Cheng D. et al., 2019). The comparatively high ROS concentration in mitochondria resulted in the transformation of PPCs from nanoparticles to fibrous nanoarchitectures. Consequently, the exposure of (KLAKLAK)_2_ to mitochondria lead to high mitochondria-targeted therapeutic efficiency without the occurrence of MDR. Interestingly, the specific delivery of drugs into mitochondria also affords a novel strategy to relief MDR.

As one of the most essential organelles, the implementation of non-drug-loaded peptide nanoassemblies for mitochondria-targeted therapy has attracted increasing attention (Jeena et al., 2017; Du et al., 2018; Li et al., 2018; Liu et al., 2018; He H. et al., 2019). Given that the elimination of cancer cells was conducted by the nanoassemblies themselves without using drugs, it undoubtedly avoids MDR. In 2016, Xu's group exploited the rationally designed peptide precursors with TPP, which targeted cancer cells and generated nanostructures *in situ* because of enzyme-instructed self-assembly (EISA, Wang et al., 2016). The cell uptake and TPP-mediated specific transportation of resulting nanostructures to mitochondria triggered the mitochondrial pathway of apoptosis without the occurrence of MDR. In another study, Ryu's team designed tripeptides by utilizing the cationic TPP as a mitochondria-targeting segment, which was denoted as Mito-FF (Jeena et al., 2019). Under the direction of TPP, Mito-FFs preferably accumulated inside mitochondria and allowed for self-assembly to activate apoptosis without the generation of MDR.

### Nucleus-Targeted Cancer Therapy

As the most essential subcellular compartment, the nucleus functions in the process of gene expression and proliferation. Given that the malignant proliferation of cells induced by gene mutation is believed to be the major cause of cancer, nucleus-targeted therapeutic supramolecular systems to hinder cellular proliferation have been intensively developed (Pan et al., 2018, see Figure 1). Up to now, various types of nuclear localization signal (NLS) peptides have been popularly employed for the specific conveyance of therapeutics to the nucleus for precise cancer treatment, containing SV40 T antigen, HIV-1 TAT peptide, and adenoviral (Pan et al., 2018).

Nucleus-targeted gene therapy by peptide-based supramolecules has long been used as one of the major approaches for cancer treatment via the nucleus-targeted transportation of therapeutic genes (Thapa and Sullivan, 2018; Cheng Y. et al., 2019; Muhammad et al., 2019). With reference to nucleus-targeted gene therapy, the major challenge is to exploit the optimal nanocarriers that are able to endure various intracellular obstacles and convey enough therapeutic genes into the nucleus. In 2018, Zhang's group generated a targeting peptide-based nanovehicle by coupling CPP cationic non-Arg (R_9_) with the tumor-targeting peptide cyclic (Arg-Gly-Asp-Phe-Lys) c(RGDfK) via click chemistry. The resulting bioconjugates could form nanocomplexes with microRNA (miRNA) and transport therapeutic genes with high specificity and efficiency (Xiao et al., 2018).

A large amount of marketed drugs are DNA-replication-related toxins, including 10-hydroxycamptothecine (HCPT), DOX, and cisplatin. In order to elicit their pharmacological activities, these drugs should be efficiently delivered into the nucleus (Han S. et al., 2015; Li et al., 2015). Considering that it is extremely hard to convey negatively charged drugs to the nucleus, Yang's group developed a nucleus-localized dual drug delivery system by the utilization of co-assembly of the positively charged cisplatin and negatively charged drug-peptide conjugate, HCPT- FFERGD (Cai et al., 2017). Using a tumor-targeted RGD peptide moiety, the negatively charged HCPT was efficiently transported to the nucleus to exert its activity. In another piece of work for HCPT delivery, Zhou's group constructed a multifunctional micellar nanoplatform, namely PECL/DA-TAT, for TAT peptide-mediated nucleus-localization of HCPT (Jing et al., 2018). As a consequence, the treatment obtained satisfying therapeutic efficiency both *in vitro* and *in vivo*.

PTT and PDT are both effective therapeutic treatments for nucleus-targeted cancer therapy due to their unique merits, including minimal drug resistance and excellent spatial selectivity. The exploitation of novel peptide-mediated supramolecular nanovehicles for nucleus-targeted PSs delivery has drawn increasing attention (Han et al., 2016a). The TAT peptide is a commonly used CPP for nucleus localization. Given that nucleus-targeted PTT can “burn” cancer cell nuclei more efficiently and afford higher therapeutic efficiency compared to other organelle-targeted PTTs, Wang's group designed a conjugate TAT-IR780 modified with the TAT peptide (Wan et al., 2020). IR780 is a NIR fluorescence for both PTT and PDT. Upon laser irradiation, TAT-IR780 efficiently destroyed genes in nucleus and induced apoptosis through both PTT and PDT. Speaking of nucleus-targeted PDT, ROS, generated by photoexcitation of nucleus-localized PS, could destroy nuclear DNA and proteins efficiently to induce cell death. In 2019, Li's team developed a self-delivery chimeric peptide, denoted as C_16_-K(PpIX)PKKKRKV-PEG_8_, for cell membrane and nucleus synergetic dual targeted PDT (Cheng H. et al., 2019d), which exhibited an improved therapeutic efficiency. The chimeric peptide consists of a hydrophobic alkyl chain (C_16_) for cell membrane targeting and an NLS peptide (PKKKRKV) for nucleus targeting.

## Conclusions and Outlooks

In this mini-review, we summarized recent advances in the field of functional peptide-decorated supramolecules for STCT, containing cell membrane-, mitochondria-, and nucleus-targeted cancer therapy. In each section, various therapeutic techniques currently used for STCT were introduced, including PTT, PDT, gene therapy, chemotherapy, and non-drug-loaded nanoassemblies. Compared with conventional targeted cancer therapy, STCT shows higher selectivity, improved sensitivity, lower dosage, and minimal adverse effects.

Due to the fact that the targeting mechanisms remain unclear, the development of lysosome-, Golgi apparatus-, and endoplasmic-reticulum (ER)-targeted nanoformulations is still challenging. Consequently, the related approaches have not received as much interest as cell-membrane-, nucleus-, and mitochondria-targeted therapies, especially for peptide-based supramolecular nanoformulations. Therefore, more research efforts should be paid in this field in the future. We believe that the discovery of new targets for lysosome, Golgi apparatus, and ER will open a new paradigm for lysosome-, Golgi apparatus- and ER-targeted therapy. STCT is still in an early stage of the development process. Despite the advantages mentioned above, there are still challenges that must be addressed for future development: (a) there is a lack of therapeutic nanoplatforms to visualize the process of STCT; (b) single therapeutic technique or single-organelle-targeted therapy often results in an insufficient therapy effect; (c) compared with cell membrane, mitochondrion, and nucleus, fewer research efforts were focused on other organelles. In view of these challenges, we propose that further research should concentrate on the following directions: (a) exploiting imaging-guided theranostic nanoplatforms for STCT; (b) developing combination/synergistic nanoplatforms for STCT; (c) concentrating more attention on the development of lysosome-, Golgi apparatus-, and endoplasmic reticulum-targeted cancer therapy. To conclude, we believe this mini review will afford helpful knowledge and new points of view in the field of functional peptide-decorated supramolecules for STCT. Under the united endeavor of researchers, clinical translation could be realized in the future.

## Author Contributions

YL and LC contributed conception and directed the work. HJ and XL contributed equally to the writing process of the first draft of the manuscript. MG revised the manuscript. All authors contributed to manuscript revision.

## Conflict of Interest

The authors declare that the research was conducted in the absence of any commercial or financial relationships that could be construed as a potential conflict of interest.
